# Reconstructive bladder surgery in genitourinary tuberculosis

**DOI:** 10.4103/0970-1591.42622

**Published:** 2008

**Authors:** Narmada Prasad Gupta, Anup Kumar, Sachit Sharma

**Affiliations:** Department of Urology, All India Institute of Medical Sciences, New Delhi - 110 029, India

**Keywords:** Caecocystoplasty, gastrocystoplasty, genitourinary tuberculosis, ileocaecocystoplasty, ileocystoplasty, orthotopic neobladder, sigmoidocystoplasty

## Abstract

Genitourinary tuberculosis (GUTB) occurs in 15-20% cases of pulmonary tuberculosis with a prevalence of 400 per 100,000 population. Reconstructive surgery for GUTB is required for cases with grossly distorted and dysfunctional anatomy that are unlikely to regress with chemotherapy alone. In the recent past, there has been a tremendous increase in the variety of reconstructive procedures for the urinary bladder, used in the management of GUTB. Augmentation cystoplasty includes the goals of increasing bladder capacity, while retaining as much of bladder as possible. Various bowel segments (from the stomach to the sigmoid colon) have been used for bladder reconstruction. The choice of material for reconstruction is purely the surgeon's prerogative--his skill, the ease, the mobility and length of mesentery (allowing bowel to reach the bladder neck without tension and maintaining an adequate blood supply). The presence or absence of concomitant reflux is of considerable importance. In the former, an ileocystoplasty with implantation of ureter to the proximal end of the isolated ileal loop and anastomosis of the distal end of the ileal loop to the bladder neck and trigone is advocated. In the latter case, the ureterovesical valve is preserved and colocystoplasty is preferred, wherein the sigmoid colon on being opened along its antimesentric border is joined to the trigone and bladder neck and then to itself to form a capacious pouch. Gastrocystoplasty reduces the risk of acidosis but is associated with complications like hypochloremic alkalosis and ‘hematuria-dysuria’ syndrome. Orthotopic neobladder reconstruction is a feasible option, suitable in cases of tubercular thimble bladder with a markedly reduced capacity (as little as 15 ml), where an augmentation alone may be associated with anastomatic narrowing or poor relief of symptoms. In this article, we review the various bladder reconstruction options used for the surgical management of GUTB, along with their indications and complications.

## INTRODUCTION

Tuberculosis (TB) has been a leading public health problem, especially in the developing nations of Southeast Asia, causing approximately three million new cases and 7,00,000 deaths every year.[1] Genitourinary tuberculosis (GUTB) - the second commonest extrathoracic form of TB, occurs due to metastatic spread of organism through bloodstream, during the initial infection. It affects males and females equally and is commonest in the fourth decade of life.[1] The kidney is usually the primary organ affected in the urinary system, and other parts of the urinary tract are involved due to direct extension. The bladder is usually affected secondary to renal tubercular involvement. Insidiousness of onset and difficulty in diagnosis may lead to a delay in treatment. This may result in serious complications such as destruction of kidney or severe involvement of the urinary bladder. Surgery continues to play a role in management of GUTB, despite the availability of effective antitubercular therapy (ATT).[2]

Bladder TB generally results in patchy cystitis due to inflammation of the urothelium by the tubercle bacilli *Mycobacterium tuberculosis*. Resultant granulomatous inflammation, caeseation necrosis, and final healing by fibrosis may lead to marked contracture of the urinary bladder within a year or so, the early disease being nonspecific. There are two types of lesions in the tubercular bladder. One, which is more common, is when the bladder due to active infection has a reduced capacity of about 150-200 ml. The other type is the true or structural bladder contracture wherein the urinary bladder has permanently lost its capacity and has little or no value as a urinary reservoir. In the initial stages before cicatrisation has taken place, the dome contracts but the trigone and bladder neck are relatively spared. Antitubercular therapy is often successful in preventing disease progression and restoring normal bladder function. But once the tubercular bladder shrinks to a very small size (reduced elasticity and compliance) - tubercular thimble bladder - the process may no longer be reversible and corrective (reconstructive) surgery in the form of augmentation must be performed to prevent/arrest kidney damage. This entails enlarging the capacity of the small, contracted, irreparably damaged bladder that causes intolerable frequency, pain, urgency and hematuria.[3]

The aims of the reconstructive surgery are - (1) enlargement of the small urinary bladder to enable the patient to retain urine for a reasonable period of time (2) restoration of function as a low-pressure (less than 30 cm of water) reservoir during storage and as a high-pressure compressor during micturition and (3) prevention of incontinence and infection that may jeopardize upper urinary tract integrity. In cases of associated vesicoureteral reflux (VUR) an increase in bladder capacity by reconstructive surgery is enough to remove the pathological changes in the upper tracts (including kidneys) caused by reflux and hence re-implantation of the ureter is unwarranted.[4] On the other hand, in cases with an intramural ureteral stricture, re-implantation is obligatory. The indication for reconstructive bladder surgery is not only tubercular vesical contracture with frequency of micturition but rather contracture with reflux and progressive hydronephrosis of kidneys. The procedure should include excision of all the diseased detrusor muscle (except trigone and bladder neck) otherwise frequency will not be relieved nor an adequate drainage established.[5]

Before any surgical intervention, a minimum of four weeks of ATT is recommended, which allows stabilization of the lesion and better planning of reconstructive surgery.[6] This period will also allow recovery of renal function in patients with deranged renal function due to outflow obstruction, if adequate temporary urinary diversion is provided.

Ideally, the material for bladder augmentation should comprise a viable graft, easily shaped, capable of distention at low pressure, accessible to endoscopy for periodic examination and not absorbing urinary constituents or secreting mucus. Metabolic and surgical complications like altered electrolyte metabolism, altered sensorium, altered hepatic metabolism, abnormal drug metabolism, calculus formation, malnutrition, growth retardation, osteomalacia, electrolyte and acid base imbalance are aspects to be looked into while choosing augmentation tissue and procedure.

## AUGMENTATION CYSTOPLASTY

This was first described in the 19^th^ century and has proved to be quite versatile for patients with tubercular affliction of the bladder.

**Indications:** Non-compliant contracted bladders where non-operative management protocols have failed.[7] The bladder loses its elasticity and compliance, leaving a capacity of less than 100 ml, in severe disease involvement. This causes intolerable symptoms like frequency, nocturia, urgency, pain and hematuria, requiring augmentation of bladder.

Renal failure is no longer a contraindication for surgery. However, the creatinine clearance of < 15 ml/minute can be considered as one of the contraindications for surgery.[8]

**Complications:** Although various procedures have proved effective, the potential for complications is also significant. Electrolyte imbalance, metabolic disturbance, excessive mucus production, calculi formation, recurrent infections and altered drug metabolism are significant complications. The use of stomach decreases the severity of some of these phenomena but may be associated with metabolic alkalosis, the ‘hematuria-dysuria’ syndrome and peptic ulceration. Long-term studies are currently uncovering the risks of growth retardation and neoplasms attributable to augmentation. The tissue used in the procedure can be a source of many complications including abscess formation, enteric fistulas, bowel obstruction, malabsorption of bile salts, fat-soluble vitamins and intractable diarrhea. Potential early and late surgical complications such as upper tract obstruction and perforation of the augmentated bladder have prompted continued search for a better biomaterial for augmentation, even though orthotopic neobladders have been successfully used in severely contracted bladders.[9]

Long-term complications of incontinence, stone formation (16%), mucus production (37g/day), metabolic derangements (16%), infection (75%), tumors or rupture of augmented bladder can be prevented by a meticulous preoperative workup including an excretory urogram (IVP), voiding cystourethrogram (VCU), cystometrogram and cystoscopy along with an authentic documentation of bladder capacity and voiding patterns. Urine specimens should be sent for routine and tubercular cultures and bladder mucosal biopsies taken to eliminate the possibility of carcinoma *in situ*. Barium enemas and sigmoidoscopies are advised for screening of colonic pathology in cases where colon is planned for use.

**Follow-up:** Regular follow-up with video urodynamics at three months followed by yearly X-Ray KUB (Kidney, Ureter and Bladder) and ultrasonographic studies with regular yearly cytoscopies after eight years are recommended.[10]

The various types of bowel segments used for augmentation cystoplasty are described below. There is no hard scientific evidence to support the use of one segment over another and one must rely on clinical experience while laboratory models and other advancements develop.

## SIGMOIDOCYSTOPLASTY (COLOCYSTOPLASTY)

This was first introduced by two Italian surgeons- Tizzoni and Foggi, who performed on a canine model- the first stage of an operation to replace the urinary bladder.[11] Lemoine used the colon for bladder augmentation for the first time.[12] This procedure seems logical as the colon (mostly the sigmoid) is comparable to the urinary bladder in as far as anatomy, sphincters and nerve supply are concerned.

**Indications:** Colonic segment is reserved for the more severely diseased and contracted bladder, while rectosigmoid proves excellent when two-thirds to three-fourths of the bladder is to be removed.[13]

**Physiology:** Micturition and defecation depend upon identical reflux mechanisms and both are under voluntary control. Micturition reflux is initiated by bladder distension with urine and defecation by passage of feces, into the rectum. Distension in either viscus serves as a stimulus to afferent nerve endings situated in their respective walls. The resultant impulses set up are conveyed through the same afferent nerve fibers derived from the parasympathetic division of the autonomic nervous system (S_2-4_) and pass to the respective walls of the bladder, sigmoid and rectum via the pelvic nerves. Motor fibers to the external urethral sphincter, external anal sphincter and to the striated muscles, in relation to the bladder and rectum, course through pudendal nerves. Higher centers for both micturition and defecation reflex are present and govern the voluntary control of respective reflexes. Keeping these neurophysiological mechanisms in mind, a segment of the sigmoid colon can be used to enlarge the capacity of the urinary bladder. The sigmoid is generally utilized in preference to the rectum because of its relative mobility. Intolerable frequency of micturition prior to operation is generally relieved after bladder reconstruction. Urination is accomplished by first emptying the original bladder followed a few moments later by sigmoidal contraction.

**Complications:** Late complications after colocystoplasty comprise chronic cystitis, persistent dysuria and frequency, along with electrolyte imbalance and metabolic disturbances.[4] Postoperative urinary drainage helps to avoid suture-line extravasation due to cystoplasty distension resulting from mucus-catheter obstruction. Temporary diversion through splinting ureteric catheter reimplantation helps.[14]

**Follow up:** Follow-up protocols incorporate a cystometry examination at one and a half months followed by a cystoscopy and cystogram at two months.[10]

## ILEOCYSTOPLASTY

Professor Antoni introduced ileocystoplasty for tubercular contracted bladder[15] though it was fashioned first by Rulkowski for extrophy bladder[16] followed by Von Mikulicz for contracted bladder.[17] The ileum has been used often - as attachment to the bladder wall by a lateral or terminal anastomosis or as a flat graft incorporated in the bladder wall.

**Indications:** The ileum provides an excellent pouch to enlarge bladder capacity when only half of the bladder is planned to be removed. It is the recommended segment for augmentation to a bladder with a fairly good capacity (200-300 ml).[14]

Male patients enjoy equal or superior success compared to female subjects.[18] Chronic renal failure was once considered a contraindication to the procedure and it was concluded that a creatinine clearance of at least 40 ml/min was mandatory. But the concept was contradicted by Kuss who successfully treated patients with a clearance of up to 15 ml/min.[19]

The results in tubercular bladder are excellent with possible factors for renal deterioration being urinary infection, renal lithiasis, vesicoureteric reflux and stenosis of various anastomoses. Low-pressure system protects the upper tracts from hydrostatic damage due to reflux, presence of vesicoenteric suture line near the trigone can lead to reflux. As far as micturition is concerned, results are better with a normal lower urinary tract. Associated lesions - prostate, bladder neck sclerosis or urethral stenosis that might interfere with the quality of micturition require complementary treatment.

**Complications:** Prolonged contact of enteric mucosa with urine leads to complications - mucus production with stone formation and infection, spontaneous perforation, metabolic disturbance, growth retardation and malignant metaplasia.[4] Associated early complications are seen in the form of pulmonary embolus, myocardial infarction, progressive azotemia, urinary and intestinal fistulas, sepsis, contracture of vesicoenteric anastomosis, ischemic fibrosis of enteric patch and wound dehiscence. Progressive azotemia, renal calculi, small bowel obstruction, ureter stricture and osteitis pubis constitute the majority of the late complications.

## CAECOCYSTOPLASTY

Couvelaire was the first to advocate the use of cecum in bladder reconstruction.[20,21] The factors that go in favor of the use of cecum for augmentation include protection afforded by the ileocecal valve,[22] its easy mobility requiring no refashioning, and its rich blood supply.[23]

It is generally suggested that as much of the bladder wall as possible should be conserved at operation, consistent with an anastomosis of 5 cm or so in diameter, making cystoplasty technically much easier and voiding better. The ileocecal valve prevents retrograde reflux of urine in the majority (64%) of cases while a reflux-preventing technique of ureteroileal anastomosis is required in cases with valve incompetence. In this context a tunnel type anastomosis rather than a cuffed nipple anastomosis proves satisfactory. High incidence of urinary fistulae is overcome by a two-layer closure with dacron while chronic retention due to obstruction at bladder neck is treated by endoscopic resection in males or urethrotomy in females.[15] Results of pressure flow videocytography confirm that the expulsive force during voiding is mostly due to abdominal wall contractions[23] and that the cystoplasty itself generates only brief low-pressure and ineffective contractions.[24] Caecocystoplasty thus provides relief of symptoms in over 90% of patients. Renal function is preserved and associated obstructive uropathy is usually relieved.

The amount of diseased bladder resected and the configuration of the cecal segment does not bear any relationship to the final surgical outcome.[25] The importance of a concurrent Y-V plasty of the bladder neck is emphasized though an efficient cystoplasty emptying is observed in 80% of patients, if detrusor resection is kept to a minimum, consistent with a wide caecovesical anastomosis.

**Complications:** No case of significant electrolyte imbalance is encountered. Common complications are enuresis, external urinary fistula, wound disruption, incisional hernia, Bladder outlet obstruction (BOO), intestinal obstruction from adhesions and contraction of cystoplasty anastomosis.[26] Operative mortality is 3.3% and the results are gratifying in selected patients after detailed voiding pattern documentation and a thorough urological study.

## ILEOCAECOCYSTOPLASTY

**Indications:** Ileocecal segments are used in those patients who have a more severely contracted and diseased bladder in which all but the trigone is removed. An antireflux anastomosis of the ureters to the ileal part of an intact ileocecal segment better protects the kidneys from back pressure and ascending pyelonephritis.

Advantages of using an ileocecal segment for cystoplasty are: (1) The technique is facilitated by vascular supply; (2) If the cystoplasty and reconstruction prove unsatisfactory, the ileal segment can be exteriorized without disturbing the original uretero-ileal anastomosis, provided they are satisfactorily and appropriately sited; (3) The ureters also can be reimplanted into the ileal segment with a natural interposing ileocecal valve mechanism to preserve the upper tracts;[13] (4) Ileum segment can be used for ureteral replacement.

## GASTROCYSTOPLASTY

Sinaiko was the first to fashion gastrocystoplasty in canine models.[27]

**Indications:** The main indications for the use of stomach in bladder reconstruction are decreased renal function and acidosis, insufficient large and small bowel and decreased mucus production.[28] Ureteric reimplantation is indicated in either reflux or stricture.

The gastrocystoplasty has several advantages, when compared with intestinal bladder augmentation, including fewer contractions, less mucus production, non-absorption of urinary electrolytes and fewer histological changes suggestive of malignancy. The technical ease of ureteral reimplantation makes the procedure suited for a severely contracted bladder due to TB with concomitant ureteral structural and renal function impairment.[29] However, use of the pedunculated wedge in reconstruction leads to transposition of more cell mass to the neobladder making urine more acidic (>3.0 ph). Also, on removal of the parietal mass from the stomach, gastric activity is reduced to a level insufficient to suppress the release of gastrin leading to severe metabolic alkalosis, harmful aciduria and hypergastrinemia. Hence antral gastrocytoplasty may be considered a better orthotopic cystoplasty wherein a simpler detubularized version proves beneficial to dampen the forceful peristaltic antral contractions compared to the more difficult muscular tunnel.[30] A further modification of the original antral technique uses the body of stomach[28] wherein the body acts as a barrier to the absorption of urinary salts and actually secretes acid. It is theoretically claimed to be better suited in patients with renal insufficiency.

**Complications:** Small capacity, absence of bladder sensation and high pressure are identified as risk factors for poor results. The removal of the parietal mass from the stomach reduces gastric activity to a level insufficient to suppress the release of gastrin leading to severe metabolic alkalosis, harmful aciduria and hypergastrinemia. The ‘hematuria-dysuria’ syndrome seen in up to 36% patients is not due to acidic urine but is also seen in patients with normal urinary ph and does not alway respond to histaminic antagonists.[31] Hypersecretion of acid after meals is generally due to a source in the body of stomach or a portion of antrum inadvertently included in the augmentation.[32]

**Follow-up:** Postoperative studies comprising urodynamic and renal function evaluation by ultrasound, serum creatinine and electrolyte measurement are obligatory.

However, with series on gastrocystoplasty being relatively small, and with the true rate of reoperation and electrolyte disturbance probably unknown, gastrocystoplasty should not be used in patients with normal bladder or urethral sensation or in those with advanced renal failure. It may still have a role in mild renal insufficiency with insensate bladder and in combination with other intestinal segments.

## ORTHOTOPIC NEOBLADDER

Though augmentation is the accepted treatment modality for tubercular thimble bladder, it may not provide symptomatic relief in markedly contracted bladders with lower urinary tract symptoms, especially the suprapubic pain. Resultant stricture formation at the anastomotic site and diverticularization of the segment may occur due to anastomosis of a bladder segment to a badly scarred end-stage tubercular bladder. Hence, an alternative surgical modality was needed that could do away with the suprapubic pain often ascribed to entrapment of nerves by cicatrisation persisting in the native bladder.[9]

**Indications:** Tubercular thimble bladder with a capacity of < 15 ml, especially when associated with significant lower tract symptoms, suprapubic pain, and lower ureteral pathology can be treated by orthotopic neobladder reconstruction as an alternative to augmentation cystoplasty in an attempt to eliminate the diseased, fibrosed, non-compliant native bladder that is left behind and fails to expand along with the augmented segment.

The procedure removes the source of symptoms, permits anastomosis to healthy tissue of the proximal urethra and addresses lower ureteral pathology at the same time. The possibility of an hourglass contracture, diverticularization and spontaneous rupture is also avoided. Lower ureteric involvement with GUTB in the form of vesico-ureteric reflux (VUR) or lower ureteral stricture is addressed at the same time. By staying close to the bladder surface, the innervation of the urethral support mechanism is preserved reducing risk of incontinence. In cases of associated advanced prostatic TB, the procedure can be modified to a nerve-sparing cystoprostatectomy with anastomosis being performed with the membranous urethra.

**Complications:** The possible complications are hypercontinence, with incomplete evacuation, nocturnal enuresis and stress incontinence, in addition to various metabolic complications of using bowel segments.[9,33]

However, longer follow-up and additional investigations are required to prove its advantages over augmentation cystoplasty.

## OUR EXPERIENCE

In the last 17 years, 241 cases of GUTB have undergone treatment at our center.[8] With a mean age of 34.6 (13-78) years there were 129 males and 112 females. Kidney was the commonest organ to be involved in 130 cases (53.94%) followed by bladder in 126 (52.28%). Ileocecocystoplasty was undertaken in 32, ileocystoplasty in 30, sigmoid colocystoplasty in 11 and bladder substitution (orthotopic neobladder) in five. In six cases, augmentation cystoplasty was done using ileal ureter, while ureteric reimplantation was undertaken in 22 cases of tubercular thimble bladder with obstructed ureter/VUR. Follow-up ranged from six months to 17 years. Early complications included spontaneous perforation of two cecum-augmented bladders, two bowel anastomosis leaks, 12 adhesive obstructions and one ileocecal anastomotic block. Late complications comprised cecovesical stenosis in three, pyonephrosis in three and progressive renal failure in 10 cases. Our study concluded that more reconstructive procedures are being performed with satisfactory outcomes, due to improved ATT and experience with use of bowel segments in urinary tract. However, it requires a long-term rigorous follow-up.[8]

## CONCLUSIONS

Reconstructive surgery has a role in the management of GUTB, despite the presence of effective ATT. The various procedures of reconstructive bladder surgery can be used according to the various indications in an individual patient. Augmentation cystoplasty offers a successful long-term solution for patients with small contracted bladders (capacity <100 ml), causing bothersome symptoms - a sequelae of genitourinary tuberculosis. It improves symptoms, prevents renal deterioration and if done for proper indications, the procedure is well tolerated resulting in gratifying long-term outcomes. Orthotopic neobladder reconstruction can be used for tubercular thimble bladder with a capacity of <15 ml, especially when associated with significant lower tract symptoms, suprapubic pain, and lower ureteral pathology, as an alternative to augmentation cystoplasty in an attempt to eliminate the diseased, fibrosed, non-compliant native bladder. However, these procedures require strict long-term follow-up of electrolytes and renal functions, infection of urine, relapse of tuberculous infection, residual urine and recurrence of hydronephrosis.
